# Identification of a 6-gene signature predicting prognosis for colorectal cancer

**DOI:** 10.1186/s12935-018-0724-7

**Published:** 2019-01-05

**Authors:** Shuguang Zuo, Gongpeng Dai, Xuequn Ren

**Affiliations:** 1 Center for Translational Medicine, Huaihe Hospital of Henan University, Kaifeng, 475001 Henan Province China; 2 Institute of Infection and Immunity, Huaihe Hospital of Henan University, Kaifeng, 475001 Henan Province China; 3 Department of General Surgery, Huaihe Hospital of Henan University, Kaifeng, 475001 Henan Province China

**Keywords:** Colorectal cancer, Differentially expressed mRNA, Prognosis, Overall survival, Gene signature, High- and low-risk

## Abstract

**Background:**

An accurate and robust gene signature is of the utmost importance in assisting oncologists to make a more accurate evaluation in clinical practice. In our study, we extracted key mRNAs significantly related to colorectal cancer (CRC) prognosis and we constructed an expression-based gene signature to predict CRC patients’ survival.

**Methods:**

mRNA expression profiles and clinicopathological data of colon adenocarcinoma (COAD) cases and rectum adenocarcinoma (READ) were collected from The Cancer Genome Atlas database to investigate gene expression alteration associated to the prognosis of CRC. Differentially expressed mRNAs (DEMs) were detected between COAD/READ and normal tissue samples. Relying on a univariate and multivariate Cox regression analyses, a mRNA panel signature was established and used for predicting the overall survival (OS) in CRC patients. Receiver operating characteristic curve was used to evaluate the prognosis performance of our model through calculating the AUC values corresponding to the 3-year and 5-year survival. To assess the performance of gene signature in the given cancer subgroups (CRC entire cohort, COAD cohort, and READ cohort), a stratified analysis was carried out according to clinical factors.

**Results:**

A total of 5341 and 5594 DEMs were collected from COAD vs. normal tissue samples, and READ vs. normal samples respectively. A univariate regression analysis for the common DEMs between COAD and READ cohorts resulted in 14 common mRNAs related to OS. The multivariate Cox regression analysis revealed that 6 of these mRNAs (EPHA6, TIMP1, IRX6, ART5, HIST3H2BB, and FOXD1) had significant prognostic value allowing the discrimination between high- and low-risk patients, implying poor and good outcomes, respectively. The stratified analysis identified 6-gene signature as an independent prognostic signature in predicting CRC patients’ survival.

**Conclusions:**

The 6-gene signature could act as an independent biomarker for survival prediction of CRC patients.

**Electronic supplementary material:**

The online version of this article (10.1186/s12935-018-0724-7) contains supplementary material, which is available to authorized users.

## Background

Colorectal cancer (CRC) is the third main cause of cancer-related death worldwide, accounting for approximately 10% of the global cancer cases [1] and it is the fourth most frequent cancer in China [2]. Rectal adenocarcinoma (READ) and colon adenocarcinoma (COAD) are two different CRC classifications based on the anatomical location. Moreover, READ shares similar molecular mechanisms with COAD [3, 4]. Despite the progresses in treatment and earlier diagnosis in the past decades, the 5-year survival rate of CRC patients is still unsatisfactory [5]. A current prognostic model according to clinical predictors such as age, gender, and tumor-node-metastasis (TNM) staging represent the conventional prognostic model for CRC in clinical practice. Nevertheless, due to the high heterogeneity of this disease, a prognosis relying on conventional clinical predictors is not precise, resulting in an inaccurate prediction of CRC patients’ survival. Thus, establishing novel predictive signatures is of great importance for a more effective treatment.

Recently, gene-prognostic signatures from gene expression analysis at messenger RNA (mRNA) level showed to provide greater accuracy in cancer prognosis than the conventional prognostic factors, which enables better individualized and more effective therapy [6, 7]. mRNAs, as important regulatory molecules, affect numerous functions, leading to many cancers including CRC [8]. Numerous works detected mRNA signatures in order to precisely predict CRC prognosis [9–11]. The over-expression of interleukin-6 mRNA is used as a predictor of relapse in colon cancer [12]. Kallikrein Related Peptidase 11 (KLK11) mRNA expression predicts poor disease-free survival (DFS) and overall survival (OS) in COAD patients [13]. Matrix Metallopeptidase 9 (MMP-9) is an important signature for postoperative prognosis and risk of metastases in CRC patients [14]. Another study showed that gastrin releasing peptide (GRP) can better predict the prognosis of CRC patients and distant metastasis with good specificity and sensitivity [15]. Li et al. [16] suggested that GRP and transmembrane protein 37 (TMEM37) may act as independent DFS prognostic genes in colon cancer. Moreover, a meta-analysis was conducted to evaluate the clinical usefulness of several published prognostic gene signatures in CRC [17]. Thus, the establishment of novel CRC-associated gene prognostic signatures to guide patients’ prognostic stratification and personalized therapy is urgently needed. Of note, investigators paid more attention in identifying a single cancer-associated mRNA as a candidate signature, which cannot be effective in predicting prognosis and choosing an individualized treatment. Hence, identification of a more accurate and robust mRNA panel signature that can predict CRC prognosis is of considerable importance.

In a previous recent study, Sun et al. [18] used the gene expression profile to extract a 12-gene expression signature associated with prognosis in colon cancer patients. However, they only analyzed the COAD patients, but not READ ones. Therefore, in this work, we analyzed COAD and READ samples to identify a prognostic panel for CRC. Through the comparison of gene expression between cancer and normal tissue in The Cancer Genome Atlas (TCGA) dataset, differentially expressed mRNAs (DEMs) were found and investigated. Moreover, OS prognostic analysis was performed based on all the three datasets (COAD cohort, READ cohort, and CRC entire cohort). Finally, a 6-gene expression signature associated with patient survival was established by exhaustively using the expression of all genes related to CRC patients from TCGA. Our results suggested that this six-gene signature could be used as a promising prognostic biomarker to effectively predict patients’ survival in CRC.

## Materials and methods

### Data source

RNA sequencing data from COAD and READ cohort consisted of 647 CRC and 51 normal samples obtained from TCGA data portal (https://tcga-data.nci.nih.gov/docs/publications/tcga/). TCGA-COAD cohort consisted of 480 COAD tissue samples and 41 adjacent normal colon tissue samples. TCGA-READ consisted of 167 READ tissue samples and 10 adjacent normal rectal tissue samples.

In addition, publicly available CRC information derived from 562 individuals with clinical follow up data were collected from the TCGA database. Among them, 419 were associated to COAD patients and 143 to READ ones. Data were downloaded from the TCGA database, thus, additional approval by an ethics committee was not required.

### Identification of DEMs in CRC

Raw data were normalized using the trimmed mean of M-values method [19], and DEMs in adjacent normal vs. COAD, and adjacent normal vs. READ samples were identified using the individual R package EdgeR (version 3.20.9) [20, 21]. DEMs were detected if ∣log2-fold change (FC)∣ > 1 and *P* value < 0.05. Volcano plots were created using the R package ggplot2 [22], and the hierarchical cluster analysis was conducted on the basis of the expression value of these DEMs using the pheatmap package (Version: 1.0.8, https://cran.r-project.org/web/packages/pheatmap/index.html) [23].

### Establishment of the predictive gene signature and risk stratification

**Fig. 1:**
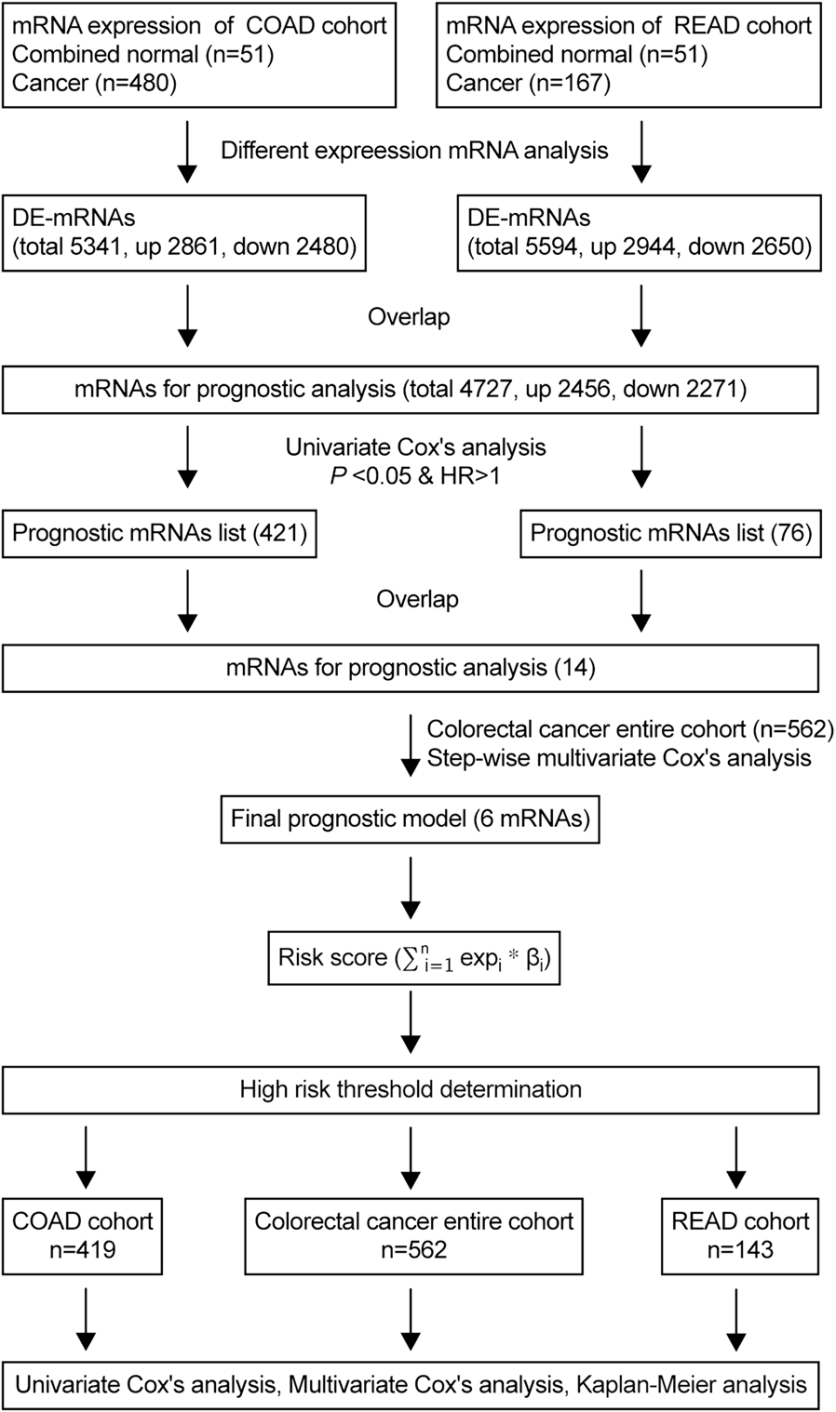
The flow chart showing the scheme of our study on mRNA prognostic signatures for CRC

The scheme of our study is illustrated in Fig. 1. The intersection of up-regulated and down-regulated mRNAs was selected for further analysis. After that, a univariate Cox proportional hazards regression analysis was used to investigate the association between DEMs expression and OS in COAD/READ patients with the purpose of evaluating which mRNAs could be potentially used as prognostic indicators for COAD/READ. Subsequently, only the common DEMs in COAD and READ with a *P*-value < 0.05 and hazard ratio (HR) > 1 were considered as candidates and subjected to a step-wise multivariate Cox regression model to extract the predictive mRNA-based model with the best explanatory and informative efficacy. Next, an mRNA-based prognostic model was used to predict the risk score for each patient as follows:

$${\text{Risk score}}\, = \,{ \exp }_{\text{mRNA1}} \,*\,\beta_{\text{mRNA1}} \, + \,{ \exp }_{\text{mRNA2}} \,*\,\beta_{\text{mRNA2}} \, + \cdots + \,{ \exp }_{\text{mRNAn}} \,*\,\beta_{\text{mRNAn}}$$

where “exp” represents the mRNA expression, and “β” is referred to the mRNA coefficient derived from the multivariate Cox regression analysis.

Based on the mRNA-based risk score equation, a risk score was obtained for each patient, and CRC patients in each cohort could be divided into high- or low-risk group using the median risk score as the threshold [24]. The receiver operating characteristic (ROC) curve was used to evaluate the sensitivity and specificity of the survival prediction according to the mRNA expression-based biomarker through analyzing the area under the curve (AUC) using the R package “survivalROC” [25]. The defining point set up by 3-year and 5-year time-dependent ROC curve analysis was employed to assess the predictive value of the risk score for time-dependent outcomes [25]. The Kaplan–Meier survival curve combined with a log-rank test was used to evaluate the differences in the patients’ survival time in the high- and low-risk group by the univariate analysis using the R package “survival”.

### Independence of the prognostic gene signature of other clinical parameters for survival prediction

Univariate Cox regression model was used to evaluate the prognostic value of the gene signature and clinical variables (including age, gender, new tumor after initial treatment, history of colon polyps, residual tumor, pathologic stage metastasis (M)/node (N)/tumor (T), tumor site, and risk score) in their relationships with patients’ OS in the CRC entire cohort, COAD cohort, and READ cohort. Then multivariate Cox regression analysis was performed to investigate whether the predictive ability of the gene signature was independent of other clinical parameters, using OS as the dependent variable, mRNA risk score and other clinical characteristics as the explanatory variables.

### Stratification analysis: prognosis performance of gene signature stratified by clinical parameters

To evaluate the prognostic performance of the gene signature in the cancer subgroups considered (CRC entire cohort, COAD cohort, and READ cohort), a stratified analysis was implemented according to clinical factors. The patients in each cohort were stratified into the two subgroups (for example, according to age, the patients were divided into ≤ 67 subgroup and > 67 subgroup), and then each subgroup was further classified into high- and low-risk group using the gene signature-based risk score. Stratification analysis was carried out using with univariate Cox regression model and the log rank test.

## Results

### DEMs identification

To evaluate the gene expression pattern in CRC, differential expression analysis was performed in COAD vs. adjacent normal samples, and READ vs. adjacent normal samples. When the criteria was set at *P *< 0.05 and |log FC| > 1, 2861 up-regulated mRNAs in COAD samples, 2944 up-regulated mRNAs in READ samples, and 2456 commonly up-regulated DEMs were found in these two cohorts. In addition, a total of 2480 down-regulated mRNAs in COAD samples and 2650 down-regulated mRNAs in READ samples were found and among them, 2271 DEMs were commonly down-regulated in the two cohorts. The volcano plot is referred to the DEMs of COAD and READ (Additional file 1: Figure S1). Hierarchical clustering results showed that COAD (Additional file 2: Figure S2) and READ (Additional file 3: Figure S3) were clearly distinguished from the adjacent normal tissue according to DEMs.

### Detection of the predictive 6-gene signature

Based on the univariate Cox regression model investigating the relationship between the 4727 DEMs (2456 commonly up-regulated DEMs plus 2271 commonly down-regulated DEMs) and the survival of patients with COAD or READ, overall 421 and 76 candidate genes were found to be significantly related to patients’ OS in the COAD and READ cohorts, respectively (*P* < 0.05 and HR > 1). Among these candidate genes, 14 were common in both.

Subsequently, with the goal of extracting the predictive signature having the best explanatory and informative efficacy, the 14 candidate mRNA were subjected to the step-wise multivariate Cox’s model, resulting in a total of 6 mRNAs identified as survival predictors, such as EPH Receptor A6 (EPHA6), Tissue Inhibitor Of Metallopeptidase Inhibitor 1 (TIMP1), Iroquois Homeobox 6 (IRX6), ADP-Ribosyltransferase 5 (ART5), Histone Cluster 3 H2B Family Member B (HIST3H2BB), and Forkhead Box D1 (FOXD1). The information related to these 6 genes is listed in Table 1.

**Table 1:** Overall information of 6 mRNAs for constructing the prognostic signature

Gene stable ID	Gene name	Gene type	Chromosome	Gene start (bp)	Gene end (bp)
ENSG00000080224	EPHA6	protein_coding	3	96,814,581	97,752,460
ENSG00000102265	TIMP1	protein_coding	X	47,582,313	47,586,789
ENSG00000159387	IRX6	protein_coding	16	55,323,760	55,330,760
ENSG00000167311	ART5	protein_coding	11	3,638,503	3,642,316
ENSG00000196890	HIST3H2BB	protein_coding	1	228,458,107	228,460,470
ENSG00000251493	FOXD1	protein_coding	5	73,444,827	73,448,527

*EPHA6* EPH receptor A6, *TIMP1* tissue inhibitor of metallopeptidase inhibitor 1, *IRX6* iroquois homeobox 6, *ART5* ADP-ribosyltransferase 5, *HIST3H2BB* histone cluster 3 H2B family member B, *FOXD1* forkhead box D1

**Fig. 2:**
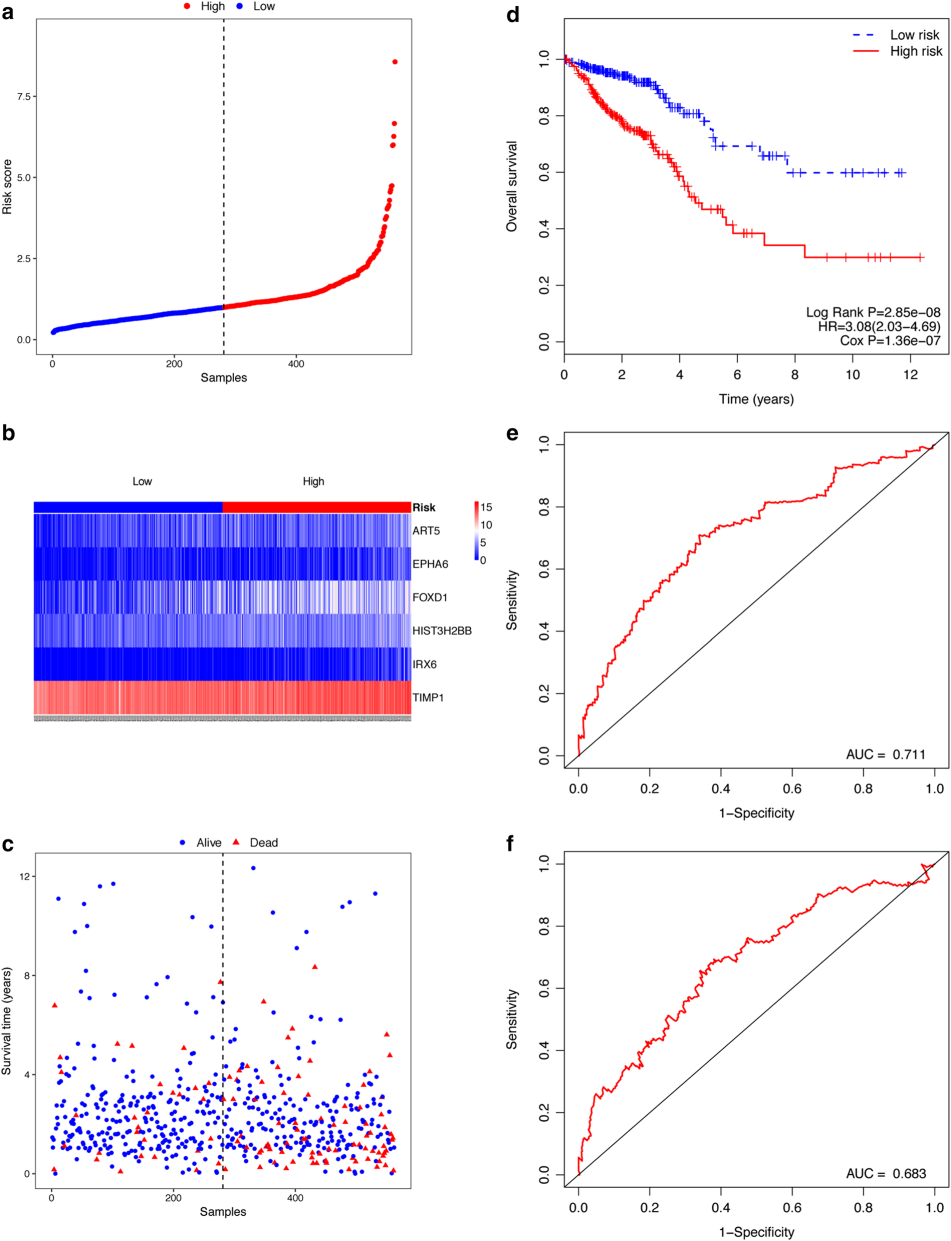
Relationship between the 6-gene signature (ART5, FOXD1, HIST3H2BB, TIPM1, EPHA6 and IRX6) and OS of patients in the CRC cohort. **a** Risk scores distribution. The blue color indicates the low-risk and the red color the high-risk. The black line indicates the median r score, which is used to separate patients into high- and low-risk group. **b** Expression pattern of the 6 prognostic genes in the high- and low-risk group. **c** Survival status. **d** Kaplan–Meier curve of OS in the high- and low-risk group. **e** ROC curve for the 3-year survival prediction by the 6-gene signature. **f** ROC curve for the 5-year survival prediction

For each patient belonging to CRC, COAD, and READ, we computed a 6-gene expression-based survival score and we assigned these scores to the high- or low-risk group based on the median risk score that was used as the cutoff point. In the CRC cohort, 562 cases were classified into high- and low-risk group using the median risk score as the threshold (Fig. 2a). Figure 2b shows the expression pattern of the 6 selected mRNAs (EPHA6, TIMP1, IRX6, ART5, HIST3H2BB, and FOXD1) in the high- and low-risk group, with the blue color representing the low expression and the red representing the high expression. The mortality rate in the high-risk group was higher than that of the low-risk group, as shown in Fig. 2c. The risk score distribution of the 6-gene, their expression and the survival status of CRC patients are shown in Fig. 2a–c respectively. The Kaplan–Meier curve with the log-rank analysis showed that the survival rate of patients in the high-risk group was lower compared to that in the low-risk group (Fig. 2d, log-rank *P* = 2.58e−08). Moreover, univariate Cox’s regression model showed that patients in the high-risk group had a significantly lower survival rate compared to that in the low-risk group (Fig. 2d, Cox P = 1.36e−07). Thus, high risk score was a poor prognostic factor for CRC patients (HR = 3.08, 95% CI = 2.03–4.69). The 3-year and 5-year survival as predicted by the risk scores are shown in Fig. 2e, f, with an AUC of 0.711 and 0.683 respectively, implying that this 6-gene signature possessed a high specificity and sensitivity in the prediction of OS.

**Fig. 3:**
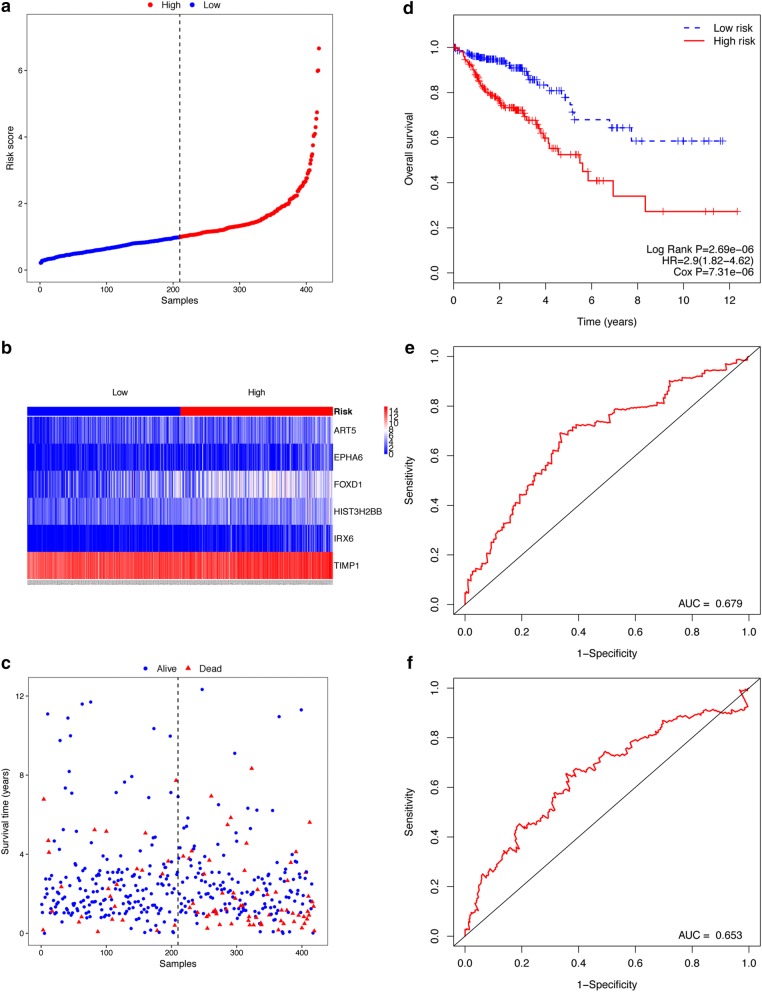
Risk score analysis of the 6-gene signature, and the association of this signature and OS of patients in the COAD cohort. **a** Risk score curve of the 6-gene signature. **b** Heatmap of the 6 prognostic genes from the COAD cohort. The color from blue to red is associated to the expression level from low to high. **c** Survival status and survival time distribution by risk scores. **d** Kaplan–Meier curve of the risk score for the OS. **e** Prognostic ability of the risk score shown by the time-dependent ROC curve for predicting the 3-years survival. **f** The prognostic ability of the risk score shown by the time-dependent ROC curve for predicting the 5-years survival

Based on the median risk score of the COAD cohort, 419 COAD patients were divided into high- and low-risk group (Fig. 3a). Figure 3b, c show the expression of the 6 mRNAs and the survival status of COAD patients, respectively. The Kaplan–Meier OS curve of the two groups showed that the patients in the high-risk group had worse prognosis than that in the low-risk group (Fig. 3d, log-rank *P* = 2.69e−06). The prognostic ability of the 6-gene signature was assessed by computing the AUC value of the ROC curve. Higher AUC corresponds to a better performance and the AUC for the 6-gene signature achieved 0.679 and 0.653 for the 3-year and 5-year survival, respectively (Fig. 3e, f), implicating the better performance of the 6-gene signature model in predicting COAD patient survival.

**Fig. 4:**
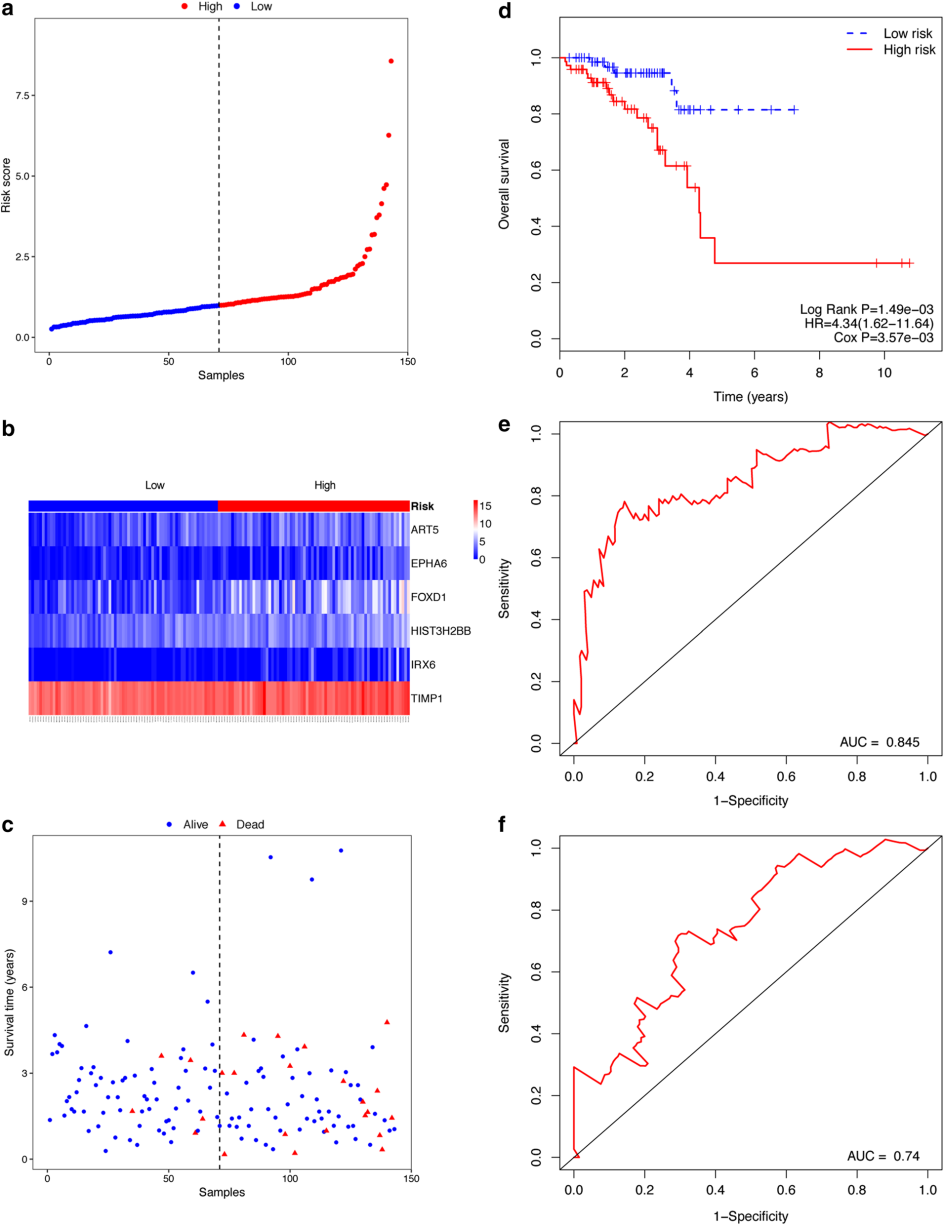
Relationship between 6-gene signature and OS of patients in the READ cohort. **a** Risk scores distribution. **b** Expression pattern of the 6 prognostic genes in the high- and low-risk group. **c** Survival status. **d** Kaplan–Meier curve of OS in the high- and low-risk group. **e** ROC curve for the 3-year survival prediction by the 6-gene signature. **f** ROC curve for the 5-year survival prediction

In the READ cohort, 143 patients were also classified into the high- and low-risk group according to the median risk score of the READ cohort. Figure 4a–c displays the risk score distribution, the expression of the 6 genes and survival status in the READ cohort. In line with the results in the CRC and COAD cohort, the patients in the high-risk group had a worse prognosis than that of the low-risk group (Fig. 4d, log-rank *P* = 1.49e−03). The ROC curve analysis achieved AUC values for the 3-year and 5-year survival of 0.845 and 0.74, respectively (Fig. 4e, f). These results confirmed that the 6-gene biomarker was able of predicting the prognosis of READ patients.

**Fig. 5:**
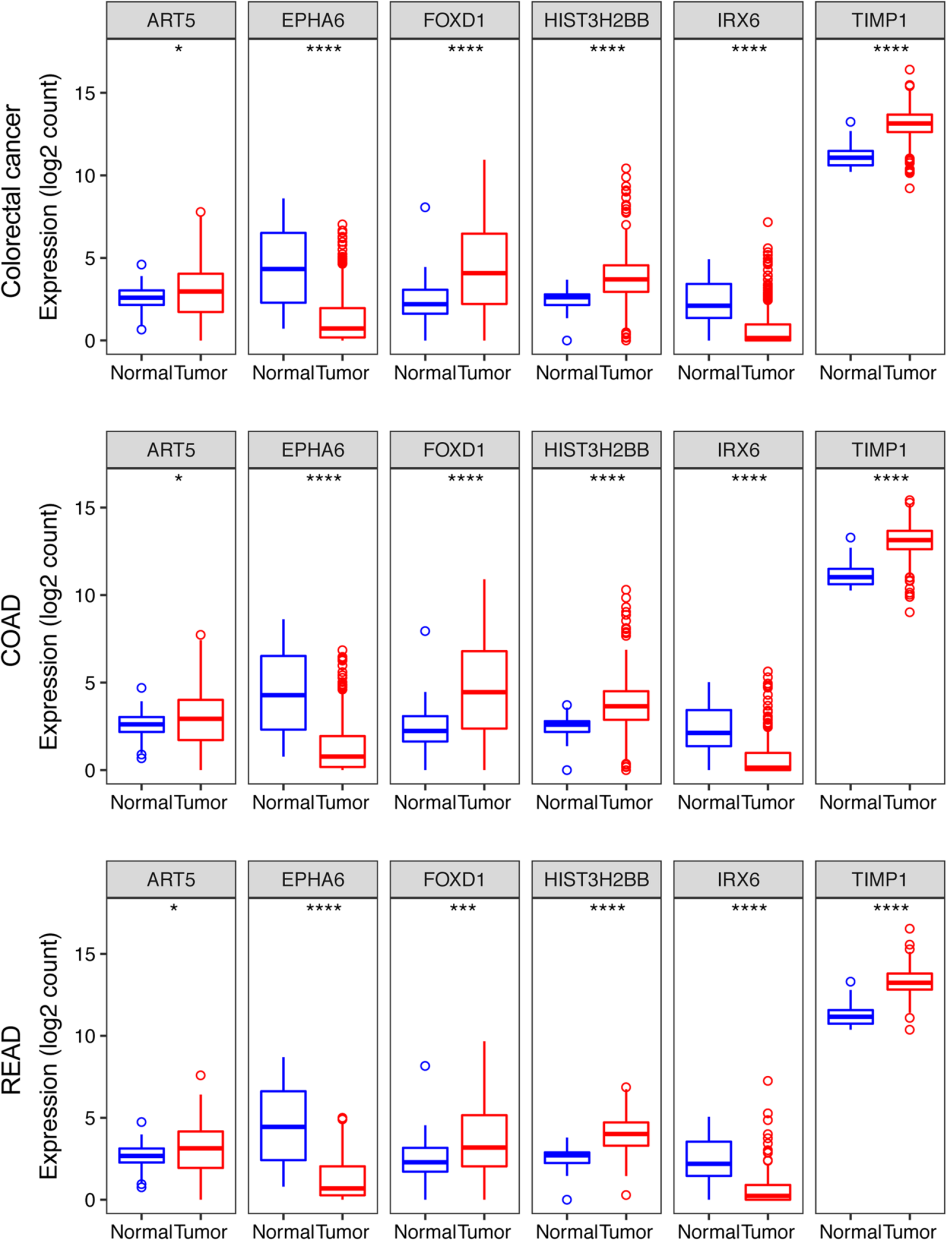
Expression pattern of the 6-gene signature (ART5, FOXD1, HIST3H2BB, TIPM1, EPHA6 and IRX6) in CRC, COAD, and READ cohort

**Fig. 6:**
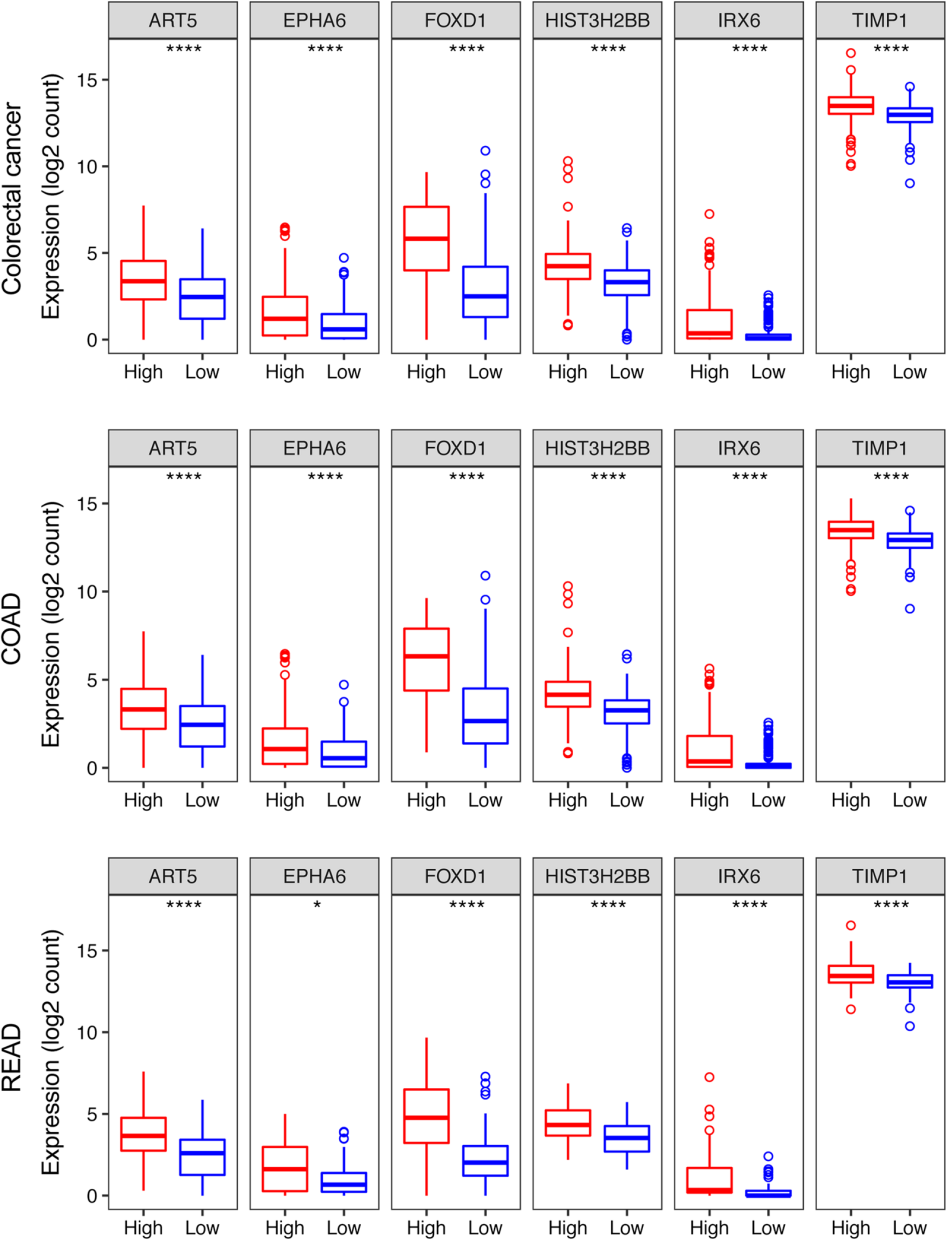
Expression pattern of the 6-gene signature (ART5, FOXD1, HIST3H2BB, TIPM1, EPHA6 and IRX6) in high- and low-risk group in each cohort

Figure 5 shows the expression patterns of all the 6 mRNAs in the three cohorts and two groups. From this figure, we found that ART5 FOXD1, HIST3H2BB, and TIMP1 expression in CRC, COAD, and READ was significantly higher than that in normal tissues (*P *< 0.05 or *P *< 0.001), while EPHA6 and IRX6 expression in CRC, COAD, and READ was lower than that in normal samples (*P *< 0.0001). The expression of all the 6 genes was significantly higher in the high-risk group compared to the low-risk groups in the three cohorts (*P *< 0.05 or *P *< 0001, Fig. 6).

### Independence of the 6-gene signature of other clinical parameters for survival prediction in each cohort

As shown in Table 2, univariate Cox regression model demonstrated that the 6-gene signature risk score (HR = 3.08, 95% CI = 2.03–4.69, *P* = 1.36E−07 for CRC, and HR = 2.9, 95% CI = 1.82–4.62, *P* = 7.31E−06 for COAD), age (HR = 1.03, 95% CI = 1.01–1.04, *P* = 7.99E−04 for CRC, and HR = 1.02, 95% CI = 1.00–1.04, *P* = 4.20E−02 for COAD), new tumor after initial treatment (HR = 2.52, 95% CI = 1.70–3.74, *P* = 4.26E−06 for CRC, and HR = 2.53, 95% CI = 1.63–3.93, *P* = 3.86–05 for COAD), residual tumor (HR = 3.96, 95% CI = 2.26–6.96, *P* = 1.65E−06 for CRC, and HR = 3.81, 95% CI = 1.89–7.68, *P* = 1.81E−04 for COAD), pathologic stage (HR = 3.17, 95% CI = 2.09–4.79, *P* = 4.83E−08 for CRC, and HR = 3.07, 95% CI = 1.95–4.85, *P* = 1.41E−06 for COAD), stage M (HR = 4.51, 95% CI = 2.95–6.89, *P* = 3.92E−12 for CRC, and HR = 4.69, 95% CI = 2.87–7.66, *P* = 6.55E−10 for COAD), stage N (HR = 2.90, 95% CI = 1.96–4.30, *P* = 1.04E−07 for CRC, and HR = 2.86, 95% CI = 1.85–4.42, *P* = 2.21E−06 for COAD), and stage T (HR = 2.18, 95% CI = 1.13–4.18, *P* = 1.94E−02 for CRC, and HR = 2.87, 95% CI = 1.25–6.59, *P* = 1.30E−02 for COAD) were significantly related to the patients’ OS in the CRC entire cohort and COAD cohort, but other factors did not exhibit any significant correlation with OS. To further investigate whether the prognostic performance of the 6-gene signature was independent of clinical factors of CRC and COAD cases, the multivariate Cox regression analysis was performed based on the 6-gene biomarker and other clinical parameters as explanatory variables and OS as the dependent variable. As shown in Table 3, the results of multivariate Cox regression model suggested that the 6-gene signature still remained an independent factor of OS after adjustment for clinical factors, including age (HR = 1.03, 95% CI = 1.02–1.05, *P*-value = 2.19E−04 for CRC, and HR = 1.02, 95% CI = 1.01–1.04, *P* = 8.93E−03 for COAD), pathologic stage (HR = 3.26, 95% CI = 2.14–4.98, *P*-value = 4.45E−08 for CRC, and HR = 3.15, 95% CI = 1.96–5.05, *P* = 1.93E−06 for COAD), and risk score (HR = 2.37, 95% CI = 1.53–3.68, *P*-value = 1.07E−04 for CRC, and HR = 2.28, 95% CI = 1.4–3.71, *P* = 9.44E−04 for COAD). Similar results were obtained from the READ cohort. Univariate Cox regression model suggested that the 6-gene signature risk score (HR = 4.34, 95% CI = 1.62–11.64, *P* = 3.57E−03), age (HR = 1.09, 95% CI = 1.04–1.14, *P* = 2.68E−04), new tumor after initial treatment (HR = 2.53, 95% CI = 1.04–6.15, *P* = 4.03E−02), residual tumor (HR = 3.38, 95% CI = 1.23–9.28, *P* = 1.79E−02), pathologic stage (HR = 3.72, 95% CI = 1.35–10.26, *P* = 1.10E−02), stage M (HR = 4.05, 95% CI = 1.70–9.68, *P* = 1.62E−03), and stage N (HR = 3.24, 95% CI = 1.27–8.29, *P* = 1.39E−02) were significantly associated with the patients’ OS in READ cohort, but other factors did not exhibit any significant correlation with OS (Table 2). The multivariate Cox regression model implicated that the 6-gene signature was an independent factor of prognosis after adjusting for other clinical factors, including age (HR = 1.08, 95% CI = 1.03–1.14, *P* = 3.03E−03) and risk score (HR = 2.97, 95% CI = 1.07–8.22, *P*-value = 3.67E−02).

**Table 2:** Univariate analysis of clinical features and risk score

Variables	Group	Colorectal cancer			COAD			READ		
		Number	HR (95% CI)	*P* value	Number	HR (95% CI)	*P* value	Number	HR (95% CI)	*P* value
Age	≤ 67/> 67	282/280	1.03 (1.01–1.04)	7.99E−04	197/222	1.02 (1.00–1.04)	4.20E−02	85/58	1.09 (1.04–1.14)	2.68E−04
Gender	Female/male	256/306	1.11 (0.77–1.62)	5.69E−01	194/225	1.17 (0.77–1.78)	4.68E−01	62/81	0.98 (0.43–2.20)	9.54E−01
New tumor after initial treatment	No/yes	402/110	2.52 (1.70–3.74)	4.26E−06	299/81	2.53 (1.63–3.93)	3.86E−05	103/29	2.53 (1.04–6.15)	4.03E−02
History of colon polyps	No/yes	324/154	0.80 (0.48–1.33)	3.84E−01	229/124	0.76 (0.43–1.35)	3.43E−01	95/30	0.98 (0.32–2.96)	9.73E−01
Residual tumor	No/yes	407/30	3.96 (2.26–6.96)	1.65E−06	302/20	3.81 (1.89–7.68)	1.81E−04	105/10	3.38 (1.23–9.28)	1.79E−02
Pathologic stage	I–II/III–IV	299/243	3.17 (2.09–4.79)	4.83E−08	231/177	3.07 (1.95–4.85)	1.41E−06	68/66	3.72 (1.35–10.26)	1.10E−02
Stage M	M0/M1	415/76	4.51 (2.95–6.89)	3.92E−12	309/55	4.69 (2.87–7.66)	6.55E−10	106/21	4.05 (1.70–9.68)	1.62E−03
Stage N	N0/N1–2	315/244	2.90 (1.96–4.30)	1.04E−07	244/175	2.86 (1.85–4.42)	2.21E−06	71/69	3.24 (1.27–8.29)	1.39E−02
Stage T	T1–2/T3–4	116/445	2.18 (1.13–4.18)	1.94E−02	86/333	2.87 (1.25–6.59)	1.30E−02	30/112	1.20 (0.41–3.57)	7.38E−01
Tumor tissue site	Colon/rectum	419/143	0.87 (0.55–1.36)	5.34E−01	–	–	–	–	–	–
Risk score	Low/high	281/281	3.08 (2.03–4.69)	1.36E−07	210/209	2.9 (1.82–4.62)	7.31E−06	71/72	4.34 (1.62–11.64)	3.57E−03

*HR* hazard ratio, *CI* confidence interval, *COAD* colon adenocarcinoma, *READ* rectum adenocarcinoma, *M* metastasis, *N* node, *T* tumor

**Table 3:** Multivariate analysis of clinical features and risk score

Variables	Group	Colorectal cancer			COAD			READ		
		Number	HR (95% CI)	*P* value	Number	HR (95% CI)	*P* value	Number	HR (95% CI)	*P* value
Age	≤ 67/> 67	282/280	1.03 (1.02–1.05)	2.19E−04	197/222	1.02 (1.01–1.04)	8.93E−03	85/58	1.08 (1.03–1.14)	3.03E−03
Pathologic stage	I–II/III–IV	299/243	3.26 (2.14–4.98)	4.45E−08	231/177	3.15 (1.96–5.05)	1.93E−06	68/66	2.77 (0.99–7.76)	5.24E−02
Risk score	Low/high	281/281	2.37 (1.53–3.68)	1.07E−04	210/209	2.28 (1.4–3.71)	9.44E−04	71/72	2.97 (1.07–8.22)	3.67E−02

*HR* hazard ratio, *CI* confidence interval, *COAD* colon adenocarcinoma, *READ* rectum adenocarcinoma

In summary, the 6-gene risk score was an independent adverse prognostic factor for the three cohorts.

### Stratification analysis: prognostic value of 6-gene signature stratified by clinical parameters

**Fig. 7:**
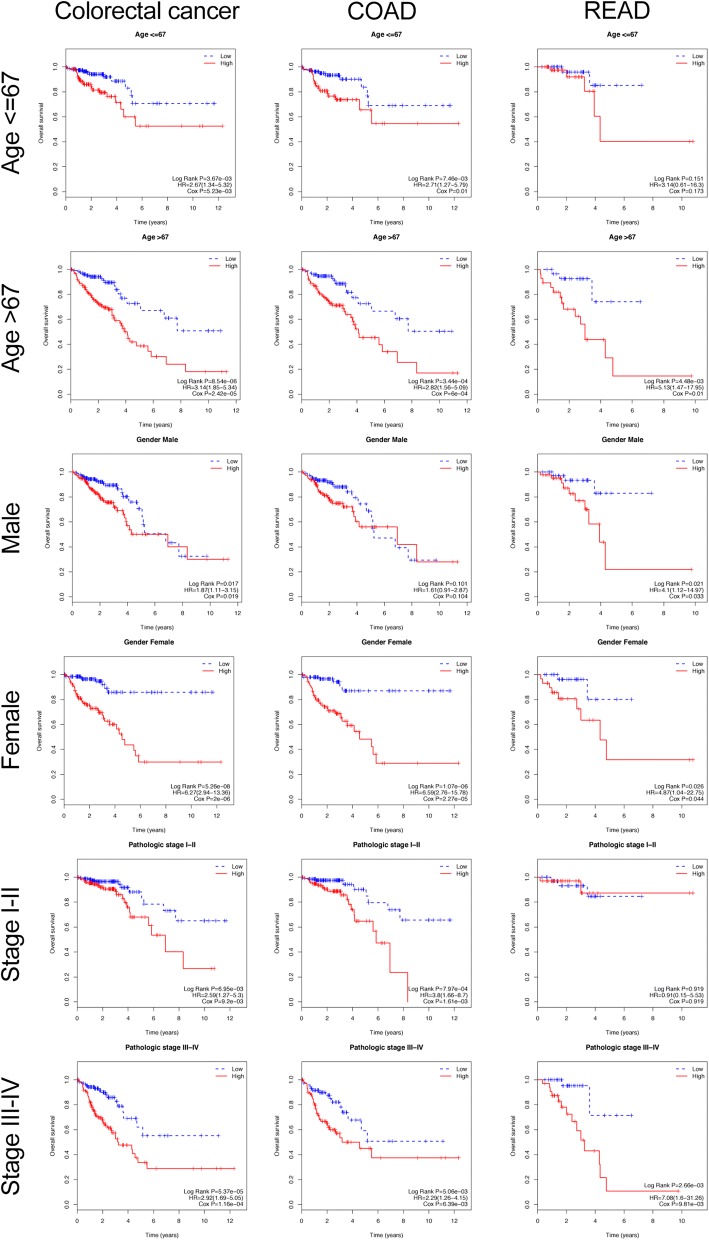
Prognostic performance of different clinical factors for survival of CRC, COAD, and READ patients. Kaplan–Meier curve of the OS in the age, gender and stage cohort stratified by 6-gene signature

With the goal of evaluating the prognostic performance of the 6-gene signature, the patients in each cohort were firstly stratified into two subgroups based on clinical parameters (such as age (≤ 67/> 67), gender (Female/Male), and stage (I–II/III–IV)), and then each subgroup was further classified into high- and low-risk group using the 6-gene signature. In all the subgroups of the CRC entire cohort, patients in the high-risk group had a significantly shorter survival time than that in the low-risk group (Fig. 7, *P* < 0.05), suggesting that the 6-gene risk score was an adverse prognostic factor in CRC. In the subgroups of the COAD cohort (except the male subgroup), patients in the high-risk group had also a significantly poorer prognosis compared to that of the patients in the low-risk group (Fig. 7, *P* < 0.05), demonstrating that the 6-gene risk score can predict the survival status in COAD patients. In the READ cohort, except the subgroup of stage I–II, patients of the other subgroups in the high-risk group had also a significantly poorer prognosis compared to that of the patients in the low-risk group (Fig. 7, *P* < 0.05), implying that the 6-gene risk score was an adverse prognostic indicator able to predict the survival status in READ patients. Combining all these results, the 6-gene signature was an independent predictor of other clinical factors for predicting survival in CRC patients.

## Discussion

Clinical predictors such as age, gender, and TNM stage are the appropriate reference for a prognostic prediction in the patients with CRC. Nevertheless, predicting capacity needs to be further improved because of the high heterogeneity of this disease. Thus, the detection of prognostic biomarkers in CRC is urgently needed. mRNA prognostic biomarkers can robustly predict the survival status of CRC patients [26–28]. Moreover, the combination of several signatures has a better predictive ability than a single biomarker. Hence, in the current study, we identified a 6-mRNA panel signature (ART5, FOXD1, HIST3H2BB, TIMP1, EPHA6 and IRX6) for CRC after the univariate and multivariate Cox proportional hazards regression analysis on the mRNA expression profile from the CRC, COAD, and READ patients on the basis of the data collected from the TCGA database. Then, a risk score was obtained by combining the 6 mRNAs and this 6-gene signature was able to independently predict OS in CRC, COAD and READ patients, further demonstrating that the risk score developed from these 6 mRNAs might be an indicator for CRC patients survival in clinical practice.

The mRNA ART5 was one of the six prognosis-related mRNAs in our study. ADP-ribosylation exerts significant functions in a large amount of cellular processes, covering signal transduction, cell cycle regulation, DNA repair, and apoptosis [29]. In the process of ADP-ribosylation, ADP-ribosyltransferases (ARTs) are important catalyzing enzymes that can convert the ADP-ribose moiety of nicotinamide adenine dinucleotide to amino acids [30, 31]. ART1 is up-regulated in CT26 colon cancer cells, and ART1 silencing reduces the survival rate and increases apoptosis [32]. However, the biological role of ART5 in CRC still remains poorly defined. FOXD1 plays important roles in a great number of biological processes, for example, cell proliferation, carcinogenesis and tumor metastasis [33]. FOXD1 silencing inhibits cell proliferation in non-small cell lung cancer, while FOXD1 over-expression is related to poor prognosis in the same cancer type [34]. Han et al. [35] demonstrated that FOXD1 enhances cell proliferation of CRC cells, and is a potential valuable prognostic biomarker in CRC. A previous bioinformatics analysis revealed that HIST3H2BB expression is increased in more advanced CRC [36]. TIMP1, a member of TIMP family, is over-expressed in many cancer types and its high expression is associated to a poor prognosis. Yoshikawa et al. [37] demonstrated that TIMP1 is a useful biomarker for OS, DFS, and recurrence in patients with gastric cancer. Moreover, high TIMP1 after chemotherapy is connected with shorter OS in patients with ovarian cancer [38]. Furthermore, TIMP1 was independently related to the time to progression, and OS in patients with metastatic CRC receiving chemotherapy [39]. EPHA6 is a member of EPHs, which has a role in several physiological processes, including migration and angiogenesis [40] and it is down-regulated in CRC [41, 42]. IRX6 has not been well defined in cancer biology, particularly in CRC. As far as we know, our study is the first investigating the relationships between the 6-prognostic mRNAs with the OS time in CRC, COAD, and READ cohorts, and demonstrated a potential prognostic value of this 6-gene signature panel in CRC. Furthermore, the bioinformatics based investigation of mRNAs will be useful in future experimental studies.

Although the findings in this study might have substantial clinical significance, several disadvantages should be taken into consideration. Firstly, only samples from the TCGA database were used to build the 6-gene signature, thereby independent data from other datasets should be considered for further verification. Secondly, in vitro and in vivo studies should be considered to reveal the biological roles of these predictive mRNAs.

## Conclusion

Taken together, we established a novel 6-gene expression signature that could discriminate COAD or CRC or READ patients between poor- and good-prognostic groups through the analysis of the mRNA expression data related to a large sample from the TCGA database. This 6-gene signature panel could potentially act as an effective indicator to help identifying patients in COAD/CRC/READ cohort with high risk of poor prognosis, although the accuracy and stability of this signature panel as a prognostic classification needs further validation based on large prospective patient cohorts.

## Additional files

**Additional file 1: Figure S1.** Volcano plot showing the mRNA expression in COAD and READ, obtained using the R package ggplot2. X axis, difference in the average mRNA expression between the two groups. Y axis, log transformed false discovery rate (FDR) values. The red color is used for the up-regulated genes, while the blue one for the down-regulated genes.

**Additional file 2: Figure S2.** The 5341 DEMs in COAD. A heatmap is plotted to show DEMs expression pattern.

**Additional file 3: Figure S3.** The 5594 DEMs in READ.

## Abbreviations

CRC: colorectal cancer
COAD: colon adenocarcinoma
READ: rectum adenocarcinoma
TCGA: The Cancer Genome Atlas
DEMs: differentially expressed mRNAs
OS: overall survival
ROC: receiver operating characteristic
TNM: tumor-node-metastasis
DFS: disease-free survival
AUC: area under the curve
EPHA6: EPH receptor A6
TIMP1: tissue inhibitor of metallopeptidase inhibitor 1
IRX6: iroquois homeobox 6
ART5: ADP-ribosyltransferase 5
HIST3H2BB: histone cluster 3 H2B family member B
FOXD1: forkhead box D1
