# Supplementary figures and images for: Identification of a 6-gene signature predicting prognosis for colorectal cancer
Source: Cancer Cell Int. 2019 Jan 5;19:6. doi: 10.1186/s12935-018-0724-7 (PMC6321660; doi:10.1186/s12935-018-0724-7)

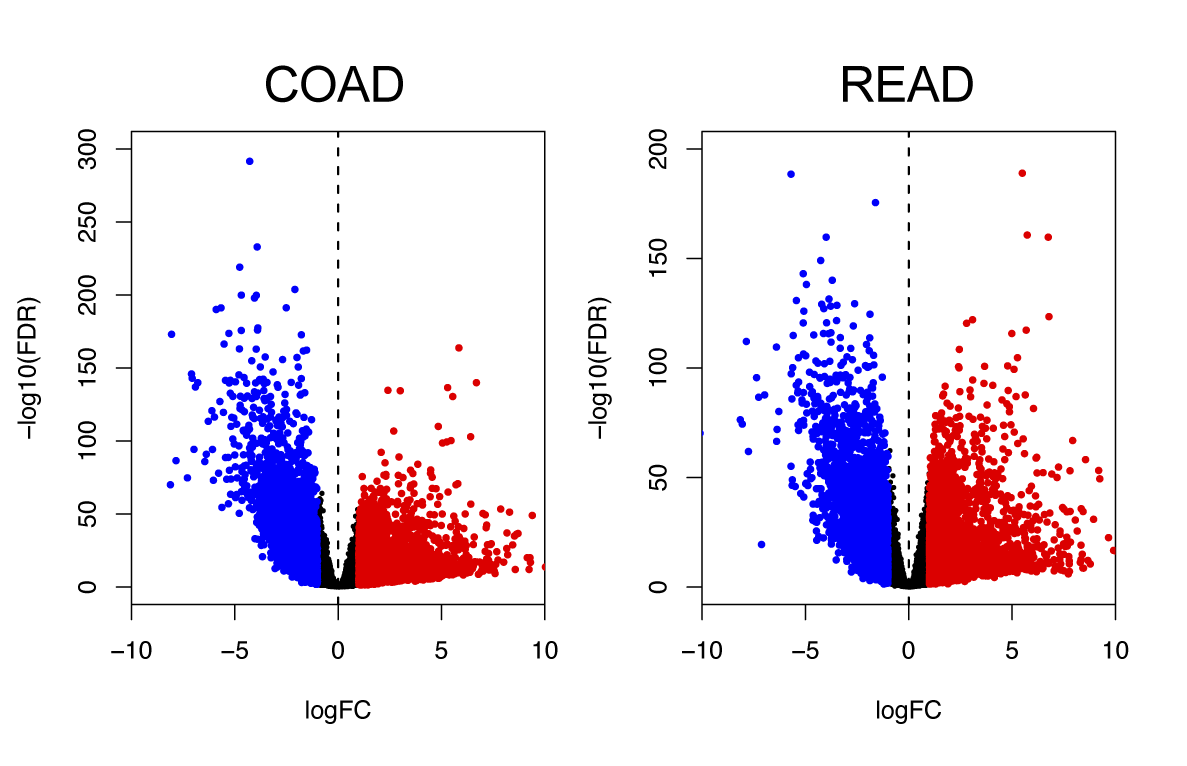

Supplement: Supplementary file 1 — Additional file 1: Figure S1. Volcano plot showing the mRNA expression in COAD and READ, obtained using the R package ggplot2. X axis, difference in the average mRNA expression between the two groups. Y axis, log transformed false discovery rate (FDR) values. The red color is used for the up-regulated genes, while the blue one for the down-regulated genes. [file 12935_2018_724_MOESM1_ESM.tif]

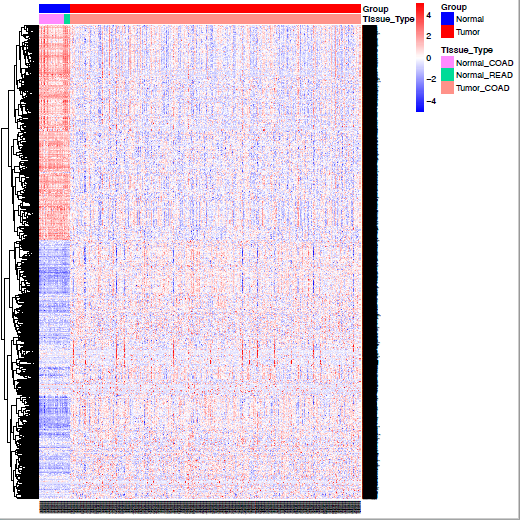

Supplement: Supplementary file 2 — Additional file 2: Figure S2. The 5341 DEMs in COAD. A heatmap is plotted to show DEMs expression pattern. [file 12935_2018_724_MOESM2_ESM.tif]

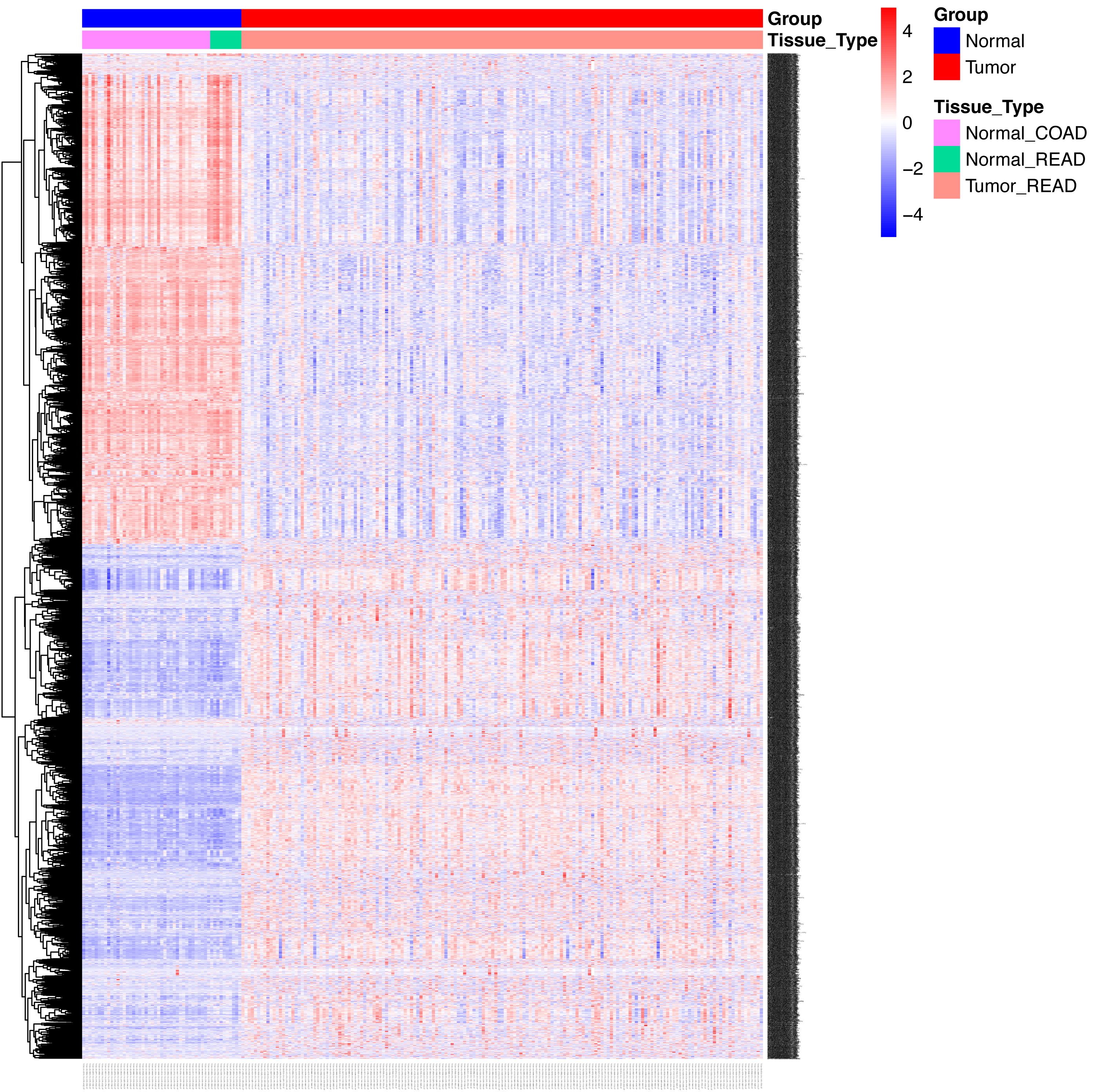

Supplement: Supplementary file 3 — Additional file 3: Figure S3. The 5594 DEMs in READ. [file 12935_2018_724_MOESM3_ESM.tif]
